# Molecular subtypes of oropharyngeal cancer show distinct immune microenvironment related with immune checkpoint blockade response

**DOI:** 10.1038/s41416-020-0796-8

**Published:** 2020-04-01

**Authors:** Min Hwan Kim, Jae-Hwan Kim, Ji Min Lee, Jae Woo Choi, Dongmin Jung, Hojin Cho, Hyundeok Kang, Min Hee Hong, Su Jin Heo, Se Heon Kim, Eun Chang Choi, Da Hee Kim, Young Min Park, Sangwoo Kim, Sun Och Yoon, Yoon Woo Koh, Byoung Chul Cho, Hye Ryun Kim

**Affiliations:** 1 Department of Internal Medicine, Division of Medical Oncology, Yonsei Cancer Center, Yonsei University College of Medicine, Seoul, Republic of Korea; 2 Institute for Cancer Research, Yonsei Cancer Center, Yonsei University College of Medicine, Seoul, Republic of Korea; 3 Brain Korea 21 PLUS Project for Medical Science, Yonsei University College of Medicine, Seoul, Republic of Korea; 4 Department of Pharmacology, Yonsei University College of Medicine, Seoul, Republic of Korea; 5 Department of Nuclear Medicine, Yonsei University College of Medicine, Seoul, Republic of Korea; 6 Department of Biomedical Systems Informatics, Brain Korea 21 PLUS Project for Medical Science, Yonsei University College of Medicine, Seoul, Korea; 7 Department of Otorhinolaryngology, Yonsei University College of Medicine, Seoul, Republic of Korea; 8 Department of Pathology, Yonsei University College of Medicine, Seoul, Republic of Korea

**Keywords:** Tumour immunology, Cancer microenvironment, Head and neck cancer

## Abstract

**Background:**

Oropharyngeal cancer (OPC) exhibits diverse immunological properties; however, their implications for immunotherapy are unknown.

**Methods:**

We analysed 37 surgically resected and nine recurrent or metastatic anti-programmed death-1 (PD-1)/programmed death-ligand 1 (PD-L1)-treated OPC tumours. OPCs were classified into immune-rich (IR), mesenchymal (MS) and xenobiotic (XB) subtypes based on RNA-sequencing data.

**Results:**

All IR type tumours were human papillomavirus (HPV) positive, most XB types were HPV negative, and MS types showed mixed HPV status. The IR type showed an enriched T cell exhaustion signature with PD-1^+^ CD8^+^ T cells and type I macrophages infiltrating the tumour nest on multiplex immunohistochemistry. The MS type showed an exclusion of CD8^+^ T cells from the tumour nest and high MS and tumour growth factor-β signatures. The XB type showed scant CD8^+^ T cell infiltration and focal CD73 expression. The IR type was associated with a favourable response signature during anti-PD-1/PD-L1 therapy and showed a high APOBEC mutation signature, whereas the MS and XB types showed resistance signature upregulation. Among anti-PD-1/PD-L1-treated OPC patients, the IR type showed a favourable clinical response (3/4 patients), whereas the XB type showed early progression (3/3 patients).

**Conclusion:**

Our analysis classified OPCs into three subtypes with distinct immune microenvironments that are potentially related to the response to anti-PD-1/PD-L1 therapy.

## Background

Immune checkpoint blockade exerts profound anti-tumour effects in many cancer types; however, the efficacy of immune checkpoint blockades is greatly affected by the tumour microenvironment. Previous studies have classified tumours into “immune-rich” and “immune desert” types based on their microenvironment.^1^ In addition, a recent study has also described an “immune-exclusion” type that is derived from tumour growth factor-β (TGF-β) activation with a large stromal component.^2^ However, the molecular determinants of the tumour immune microenvironment are largely unknown, and numerous tumoural and host factors, such as tumour mutational burden (TMB),^3^ oncogene mutations, host HLA type^4^ and microbiota^5^ are expected to influence of the tumour microenvironment.

Recurrent and metastatic head and neck squamous cell carcinoma (HNSCC) is incurable and causes frequent functional disability and high mortality.^6^ Clinical trials have demonstrated promising clinical efficacy of anti-programmed death-1 (PD-1) therapy in HNSCCs, and nivolumab and pembrolizumab have been currently approved for HNSCC refractory to platinum-based therapy.^7–9^ However, the response rates of these immunotherapies are relatively low (13‒16%), and progression-free survival is limited in the majority of patients. Moreover, the presence of programmed death-ligand 1 (PD-L1) on tumour cells did not satisfactorily predict response, with 27% of PD-L1^+^ patients vs. 12% of PD-L1^−^ patients responding.^8^ This unsatisfactory efficacy of anti-PD-1 therapy necessitates the development of predictive biomarkers for patient selection and for effective combination immunotherapy.

HNSCC arises from heterogeneous anatomical origins from the oral cavity to the hypopharynx. Oropharyngeal cancer (OPC) constitutes a distinct clinical subset of HNSCC with a high prevalence of human papillomavirus (HPV)-associated tumorigenesis.^10,11^ OPC tumours that are HPV positive show superior survival outcomes and different molecular characteristics compared with those that are HPV negative.^10^ Notably, HPV-positive OPC tumours possess distinct immunological properties relative to HPV-negative tumours and are characterised by high levels of T lymphocyte infiltration.^12^ However, other studies have also found a significant variation in the immunologic characteristics of HPV-positive HNSCCs,^13^ and the response rate of HPV-positive HNSCC tumours to anti-PD-1/PD-L1 therapy is low.^8^ These results indicate that, in addition to HPV status, multiple molecular factors affect the immunogenicity of HNSCCs. Previous genomic studies have classified HNSCCs into different molecular subtypes;^14,15^ an enhanced understanding of the immunologic properties of each molecular subtype is predicted to contribute to the development of effective immunotherapy for HNSCCs.

In this study, we performed a comprehensive molecular profiling of OPC tumours and classified OPCs into three distinct subtypes. We found a significant difference in the immune microenvironment between the three molecular subtypes and demonstrated a correlation between anti-PD-1/PD-L1 therapy response and molecular subtype. These results suggest that immune microenvironment subtyping is beneficial for the prediction of response to immunotherapy, as well as for the design of combination immunotherapy strategies for patients with OPC.

## Methods

Also see Supplementary Methods for further information.

### Patients

This study analysed two cohorts of patients who were diagnosed with squamous cell carcinoma of the oropharynx by pathologic confirmation at the Yonsei Cancer Center. The first cohort consisted of 37 patients with local or locally advanced OPC who received curative resection between January 2011 and December 2014. The second cohort included nine recurrent or metastatic OPC patients who were administered anti-PD-1/PD-L1 blocking agents between December 2016 to October 2018.

### RNA-seq data analysis

Formalin-fixed and paraffin-embedded (FFPE) tumour specimens were obtained from all patients in the two patient cohorts, and RNA-sequencing (RNA-seq) was performed. DESeq2 package was used for differentially expressed gene (DEG) analysis. The RNA-seq data from OPC tumours were subjected to two-dimensional t-distributed stochastic neighbour embedding (t-SNE) plotting using Rtsne package. Unsupervised clustering of the t-SNE result was performed using *k*-means clustering. The clustering yielded three molecular subtypes of OPC tumours.

### Targeted panel sequencing

Genomic DNA was purified from FFPE tissues and peripheral blood mononuclear cells from patients’ blood for targeted sequencing. As previously described,^16^ the 244 head and neck cancer-related genes were incorporated into our targeted sequencing panel. To capture the genomic regions of these genes, the customised SureSelectXT Target Enrichment library generation kit (Agilent, Santa Clara, CA, USA) was used.

### Multiplex IHC image analysis

For multiplex immunofluorescence stain of the sections, the automated staining system (BOND Rx, Leica Biosystems) was used with the Opal 7-colour automation immunohistochemistry (IHC) kit (PerkinElmer). The multi-spectral images for analyses of tissue contents were generated from whole-slide scan images by Vectra Polaris (PerkinElmer) and Phenochart software (PerkinElmer). Immune cells were quantified by cell counts per mm^2^ in both the tumour nest and stroma. Membranous PD-L1 or CD73 expression on tumour cells was determined by tumour proportion score using the InForm software.

## Results

### Molecular subtypes of surgically resected OPC tumours

**Fig. 1:**
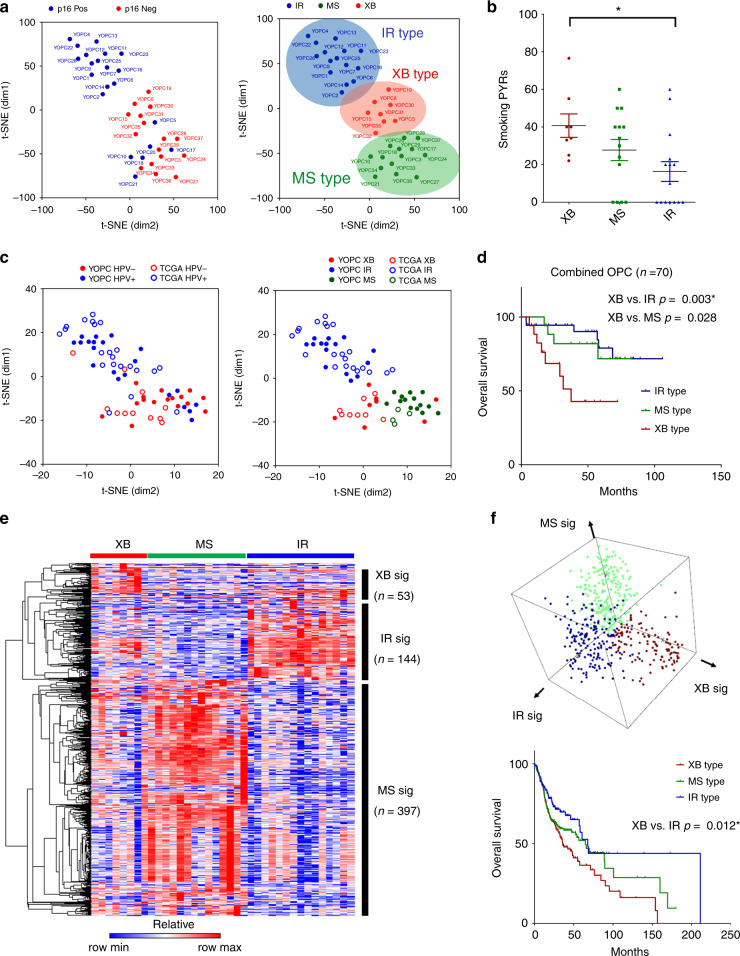
Three molecular subtypes of OPC identified by RNA-seq analysis of tumours show different survival outcomes. **a** The two-dimensional t-SNE plot of RNA-seq data from 37 surgically resected OPC tumours (YOPC) with p16 IHC status. The *k*-means clustering analysis classifies the tumours into three subtypes: immune-rich (IR), mesenchymal (MS) and classic (CL) type. The patient number (YOPC) corresponds to the plots. **b** Comparison of smoking doses of patients among three subtypes. The smoking dose in pack-years (PYR) was compared using Kruskal–Wallis test with post hoc analysis using Dunn’s multiple comparison test. The difference between XB and IR type OPCs was statistically significant (*p* < 0.05) in the post hoc analysis and indicated with the asterisk (*p < 0.05). **c** The t-SNE plotting for OPC tumours of the combined cohort, which consists of 37 OPC tumours from patients at the Yonsei Cancer Center (YOPC) and 33 HNSC TCGA tumours of oropharyngeal origin (the soft palate, the base of tongue, the tonsils and the side and back wall of the throat). The subtype of TCGA OPC tumours was determined by *k*-means clustering. **d** The Kaplan–Meier curves for overall survival of OPC patients in the combined cohort. The patient survival was compared using log-rank test. The difference between XB and IR type OPCs was statistically significant (*p* < 0.167) after the Bonferroni post hoc correction and indicated with the asterisk (*). **e** The heatmap of significantly altered genes among three subtypes (log_2_ fold change ≥2 and *p* value < 0.05). The CL-, IR- and MS-specific gene signatures were selected according to their expression in each subtype. **f** The three-dimensional plotting and *k*-means clustering analysis using gene set enrichment score of each subtype signature derived from **e** calculated by GSVA algorithm in 518 HNSC TCGA patients (upper panel). The Kaplan–Meier curves for overall survival of 518 HNSC TCGA patients according to subtype classification (lower panel). The difference between XB type vs. IR type OPCs was statistically significant (*p* < 0.167) after the Bonferroni post hoc correction. Throughout the figures, data are mean and SEM.

We performed unsupervised clustering analysis of RNA-seq data of 37 surgically resected OPC tumours (YOPC) for molecular subtyping. After filtering out genes with low expression, the normalised expression values of 18,862 genes were subjected to t-SNE analysis followed by *k*-means clustering. The clustering revealed three distinct molecular subtypes of OPCs (Fig. 1a). The subtypes were named as “immune-rich” type (IR type), “xenobiotic” (XB type) and “mesenchymal” (MS type), according to their molecular nature as described later. The HPV status of tumours was evaluated using p16 IHC analysis and validated by HPV genotyping polymerase chain reaction (PCR). We detected HPV DNA in 20 out of 21 (95.2%) p16-positive OPC tumours, and HPV PCR was negative in 14 out of 16 (87.5%) p16-negative OPC patients (Supplementary Table S1). The IR type consists of only HPV-positive tumours, and XB type consists of mostly HPV-negative tumours (7/8). The MS type showed mixed composition of HPV-positive and -negative tumours. The HPV PCR and p16 IHC results were concordant in all XB and IR type tumours. There were two HPV PCR (+)/p16 IHC (−) tumours and one HPV PCR (−)/p16 IHC PCR (+) tumour in MS type tumours. The total smoking dose in pack-years was higher in XB type, and MS type showed lower tumour stage than the others (Fig. 1b and Supplementary Tables S2 and S3). To generalise our molecular subtyping in an independent patient cohort, we applied our subtyping to The Cancer Genome Atlas (TCGA) HNSCC tumours with oropharyngeal origins (*n* = 33). In the combined cohort of YOPC and TCGA cohorts (total *n* = 70), the t-SNE and *k*-means clustering again identified three molecular subtypes (Fig. 1c). The HPV-positive tumours were similarly enriched in the IR type in TCGA cohort. The IR and MS type patients showed significantly better overall survival than those of XB type (Fig. 1d). We compared subtyping results between TCGA classification and our classification in our 37 YOPC tumours (Supplementary Fig. S1a) and 33 TCGA HNSCC tumours with oropharyngeal origins (Supplementary Fig. S1b). Our OPC subtyping was concordant with previous TCGA classification as follows: IR type with atypical type; XB type with classic/basal type; and MS type with mesenchymal type. However, some cases were discordant between our classification and TCGA classification, because our classification is confined to OPCs, whereas TCGA classification was derived from HNSCCs of all anatomic origins.

We identified subtype-specific gene signatures by analysing the DEGs (Fig. 1e, Supplementary Table S4, log fold change ≥2 and *p* value < 0.05) of each subtype using the RNA-seq data of 37 surgically resected OPC tumours (YOPC) and tested their relevance in the total TCGA HNSCC cohort (*n* = 518). The enrichment scores for each subtype signature in TCGA HNSCC tumour samples were calculated by gene set variation analysis (GSVA),^17^ and HNSCC tumours were classified into three subtypes (Fig. 1f). The patients’ survival was more favourable in the order of IR type, MS type and XB type, consistent with results from the combined YOPC and TCGA OPC cohort. The tumour classification using gene signature GSVA was highly concordant with t-SNE *k*-means clustering analysis in both, 37 YOPC tumours (Supplementary Fig. S2a) and combined cohort tumours (37 YOPC + 33 TCGA tumours; Supplementary Fig. S2b).

### Characterisation of gene signatures in three OPC subtypes

**Fig. 2:**
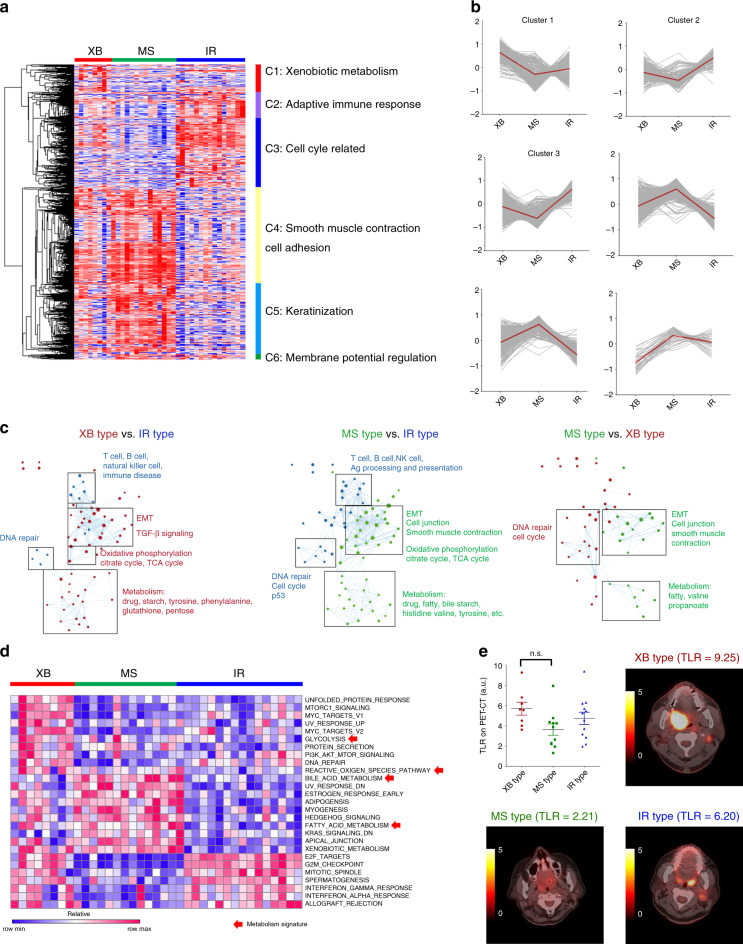
Characterisation of subtype-specific gene signatures. **a** The differentially expressed genes among the subtypes (log fold change ≥1 and *p* value < 0.05) were categorised into six clusters: xenobiotic metabolism (C1), adaptive immune response (C2), cell cycle-related (C3), smooth muscle contraction and cell adhesion (C4), keratinisation (C5) and membrane potential regulation (C6) genes in 37 surgically resected OPC tumours (YOPC). **b** Subtype-specific expression of six cluster genes. The average *z*-score expression of each gene is shown by a grey line, and the average *z*-score expression of all genes is shown by a red line. **c** The network analysis of differentially expressed genes via pair-wise comparison of each subtype using Enrichment map software in 37 YOPC tumours. **d** The heatmap showing enrichment score of hallmark gene signatures (H) in MSigDB on GSVA. Red arrows indicate the gene signatures related to metabolism in 37 YOPC tumours. **e** Tumour-to-liver uptake ratio (TLR) from F-18 FDG PET-CT scan of OPC tumours according to their subtype. The pre-operative PET-CT scan was available in 31 out of 37 OPC tumours, and the representative PET/CT images of subtypes are shown. The TLR values were compared using one-way ANOVA with Bonferroni post hoc test. The difference between XB type and MS type OPCs was statistically insignificant in the post hoc analysis. n.s., Not significant.

To characterise the molecular properties of each subtype, the significantly altered genes among subtypes (log fold change ≥1 and *p* value < 0.05) were classified into clusters 1 to 6 (from C1 to C6) by hierarchical clustering (Fig. 2a and Supplementary Table S5). The XB type showed upregulation of XB metabolism genes (C1), including *AKR1C2* and *AKR1C3*, which participate in oxidation–reduction processes in response to alcohol and cigarette exposure (Fig. 2b). The IR type showed enrichment of the adaptive immune response genes (C2), including various T cell surface markers and cell cycle-related genes (C3) associated with HPV E6/E7 oncoprotein activity. The MS type was enriched for genes related to smooth muscle contraction and cell adhesion (C4), and keratinisation (C5). Pair-wise comparison of the interactome of the three molecular subtypes showed activation of immunological pathways in IR type and epithelial–mesenchymal transition (EMT) pathways in XB and MS types (Fig. 2c). GSVA analysis using hallmark gene signatures (H) in MSigDB also indicated significant upregulation of interferon-α/γ (IFN-α/γ) response genes in IR type, unfolded protein response and glycolysis genes in XB type and myogenesis genes in MS type (Fig. 2d). The three subtypes showed differences in metabolism gene expression signatures (red arrows in Fig. 2d), and glycolysis signature was upregulated in XB type. We compared tumour metabolic rate via positron emission tomography (PET) CT scan (Fig. 2e). The XB type showed higher tumour-to-liver uptake ratio (TLR) in ^18^F-fluorodeoxyglucose positron emission tomography-computed tomography (F-18 FDG PET-CT) scan than other types, suggesting that XB type tumours have high glycolysis activity.

### Distinct immunologic properties of three subtypes

**Fig. 3:**
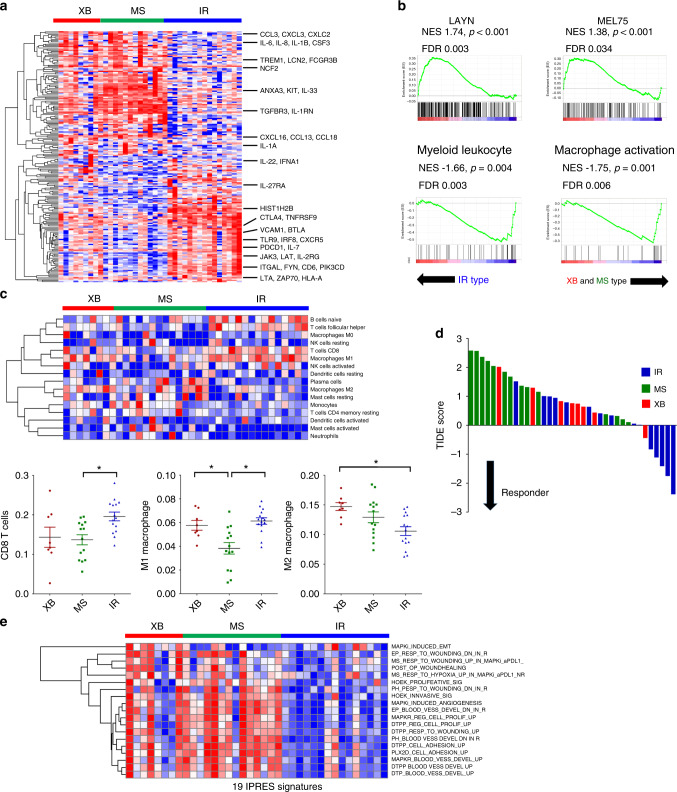
Distinct tumour immune microenvironment of OPC subtypes revealed by RNA-seq. **a** Significantly altered immune-related genes (log_2_ fold change ≥1 and *p* value < 0.05 in GO term immune response: GO:0006955) among three subtypes. The representative genes related to T cell adaptive immune response and macrophage/granulocyte activation are indicated. **b** GSEA of expression profile comparing IR type vs. XB and MS types using T cell exhaustion (LAYN and Mel75) and myeloid cell activation (GO:0002274: myeloid leucocyte activation and GO:0043030: regulation of macrophage activation) gene signatures. **c** Immune cell composition analysis using the CIBERSORT deconvolution method. The heatmap showing the relative fraction of various types of immune cells in OPC tumours (the upper panel). The dot plot showing ratio of CD8^+^ T cells, M1 macrophages and M2 macrophages in OPC tumours (the lower panels). The proportion of the cells was compared using one-way ANOVA with Bonferroni post hoc test. The statistically significant results per post hoc analysis are indicated with asterisks (*p < 0.05). **d** The waterfall plot of TIDE prediction score of 37 OPC tumours. The molecular subtype is indicated by a different colour. A low TIDE score means a high probability of response to immune checkpoint blockade therapy. **e** The heatmap of the gene set enrichment score of 19 IPRES gene sets calculated by GSVA in 37 OPC patients.

We next analysed the immunologic characteristics of OPC subtypes at the transcriptomic level. Among immune response-related genes (from GO term “immune response”, GO:0006955), 203 genes were significantly altered among the three subtypes (Fig. 3a and Supplementary Table S6, log 2 fold change ≥1 and *p* value < 0.05). Notably, genes related to T cell exhaustion (e.g. *PDCD1*, *CTLA4* and *41-BB*) were selectively upregulated in IR types, and genes related to macrophage and granulocyte infiltration and activation (e.g. *TREM1*, *CSF3* and *CCL3*) were upregulated in the XB and MS types. Accordingly, gene set enrichment analysis (GSEA) showed enrichment of T cell exhaustion signatures established by previous studies (LAYN^18^ and Mel75^19^) in IR type (Fig. 3b). In contrast, the myeloid cell- and macrophage-related signatures were enriched in XB and MS types in comparison with the IR type. Next, we employed the CIBERSORT algorithm^20^ to estimate the composition of tumour-infiltrating immune cells (Fig. 3c). The number of activated CD8^+^ T cells was significantly higher in IR type, and that of type I macrophage was lower in the MS type. Conversely, type II macrophage infiltration was significantly lower in the IR type. The estimated NK cell and B cell infiltration did not differ among the three subtypes. IR type tumours were also predicted to be responsive to tumour immunotherapy, as indicated by tumour immune dysfunction and exclusion (TIDE) scores (Fig. 3d).^21^ These results collectively indicate IR type as highly immunogenic and responsive to anti-PD-1/PD-L1 blockade. Recently, Hugo et al.^22^ suggested the 19 gene signatures to be innate anti-PD-1 resistance (IPRES) signatures. Of note, 18 of 19 signatures were upregulated in XB and MS type tumours compared with those in IR type tumours (Fig. 3e). These data support that XB and MS types may exhibit resistance to anti-PD-1/PD-L1 therapy.

### Multiplex immunohistochemistry reveals distinct immune microenvironment of the subtypes

**Fig. 4:**
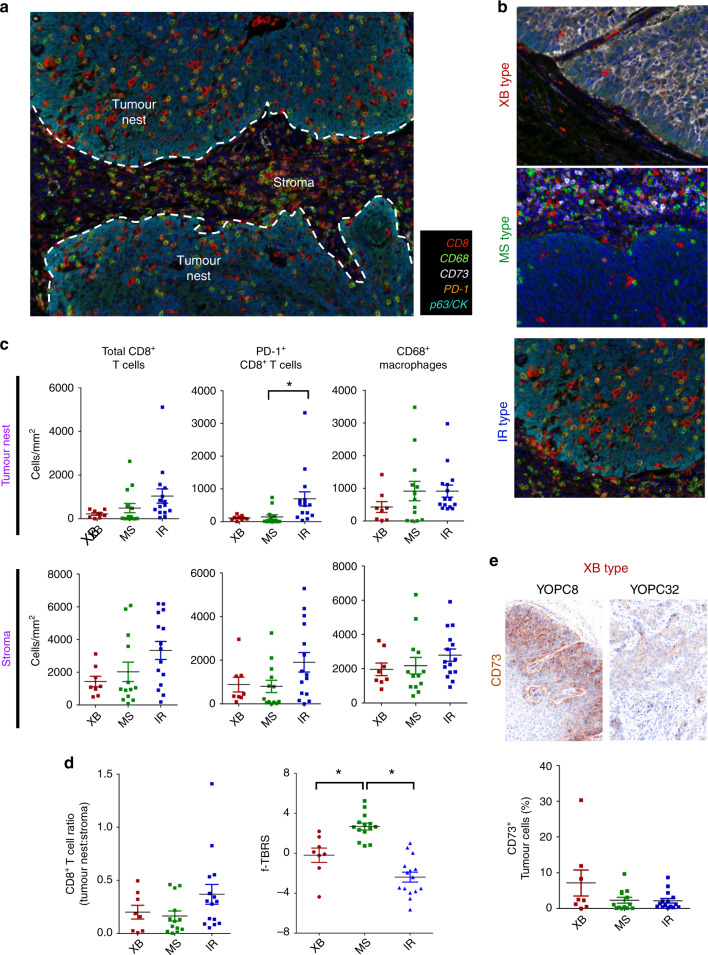
Distinct immune microenvironment of OPC subtypes revealed by multiplex IHC. **a** The representative multiplex IHC image of OPC tumour. The representative tumour nest and stroma sites were selected, and the density of CD8^+^ T cells and CD68^+^ macrophages, as well as the proportion of CD73^+^ tumour cells were counted both in tumour nests and stroma. **b** The representative multiplex IHC images of subtypes of OPC tumours. **c** The dot plots of immune cell densities of OPC tumours, total CD8^+^ T cells, PD-1^+^CD8^+^ T cells and CD68^+^ macrophages both in tumour nests and stroma. **d** The ratio of CD8^+^ T cell density in tumour nest to CD8^+^ T cell density in stroma in the three subtypes (left panel). The f-TBRS score of the three subtypes in 37 YOPC tumours (right panel). **e** The representative CD73 IHC image of two XB type tumours (YOPC8 and YOPC32, upper panel). The dot plot of the proportion of CD73^+^ tumour cells in the subtypes (lower panel). The cell densities (**c**) and f-TBRS (**d**) were compared using one-way ANOVA with Bonferroni post hoc test. The statistically significant results per post hoc analysis are indicated with asterisks (*p < 0.05).

To directly examine the immune microenvironment of the three OPC subtypes, we performed multiplex IHC analysis using the Vectra imaging system in 36 of 37 OPC tumours (Fig. 4a, tumour tissue for IHC was unavailable for one patient). The tumour-infiltrating immune cell densities, including CD8^+^ T cells, PD-1^+^CD8^+^ T cells and CD68^+^ macrophages were measured both in tumour nests and stroma using automated imaging analysis. Consistent with RNA-seq analysis, the IR type showed several PD-1^+^CD8^+^ T cells and CD68^+^ type I macrophages infiltrating the tumour nest, while the XB and MS types showed scant CD8^+^ T cell infiltration in tumour nests (Fig. 4b). The density of total CD8^+^ T cells and PD-1^+^CD8^+^ T cells was higher in IR type tumours than in the other types in both tumour nests and stroma (Fig. 4c). The MS type tumours showed higher total CD8^+^ T cell density than XB type, but lower tumour nest-to-stroma CD8^+^ T cell ratio than the IR type, suggesting exclusion of CD8^+^ T cells from tumour nests (Fig. 4d). Previous studies reported an immune-exclusion phenotype with enhanced TGF-β activity and high stromal component, and the MS type tumours showed a significantly higher TGF-β-response signature (TBRS) score^2^ (Fig. 4d). We also found upregulation of CD73, which plays an important immunosuppressive role in CD39/C73-dependent adenosine pathways, in tumour cells in two XB type tumours (Fig. 4e).

### Genetic alterations and TMB of subtypes

**Fig. 5:**
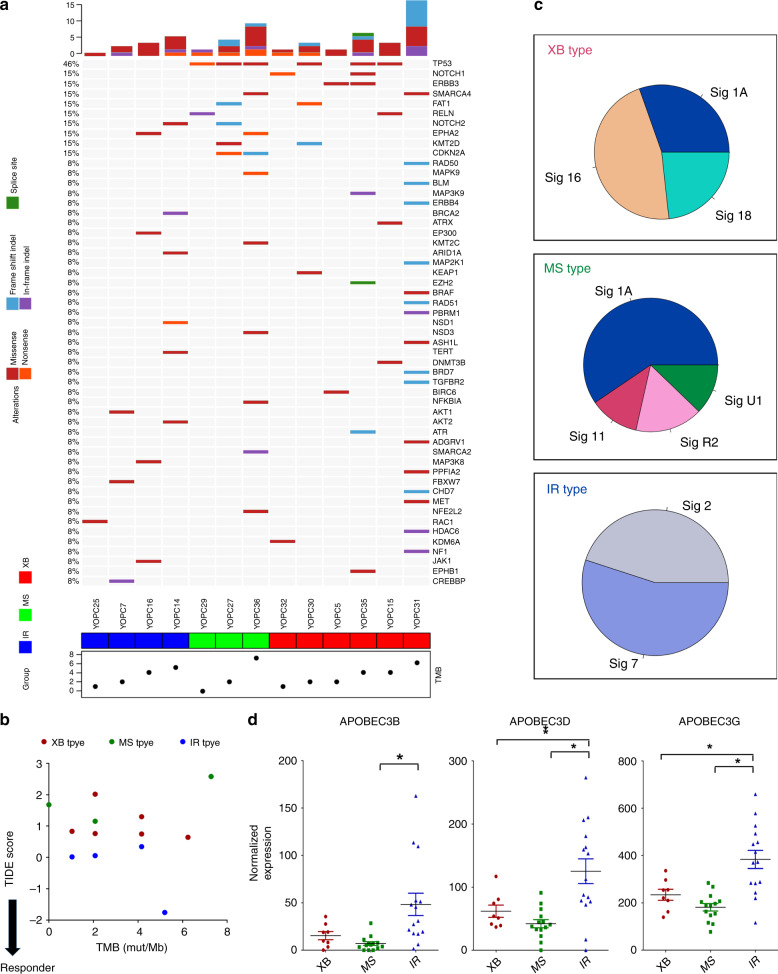
Analysis of subtype-specific genetic properties by next-generation sequencing of OPC tumour DNA. **a** The oncomap showing genetic alterations of OPC tumours. The tumour-blood-matched targeted panel sequencing was performed in 13 out 37 OPC patients with available samples. The tumour subtype is indicated at the bottom of the map and the tumour mutation burden (TMB, non-synonymous mutations/Mb) was plotted. **b** The plot showing correlation between tumour mutation burden and TIDE score in OPC tumours. **c** Pie charts showing the proportion of COSMIC mutational signatures in XB type, MS type and IR type of OPC tumours. **b** The dot plot comparing the expression level of APOBEC family genes, *APOBEC3B*, *APOBEC3D* and *APOBEC3G*, in the three subtypes. The normalised expression level on RNA-seq was compared by unpaired *t* test. The expression values were compared using one-way ANOVA with Bonferroni post hoc test. The statistically significant results per post hoc analysis are indicated with asterisks (*p < 0.05).

We next compared genetic alterations of OPC molecular subtypes using targeted panel next-generation sequencing (NGS). We performed NGS analysis of FFPE tumour samples with available matched normal blood samples in 13 of 37 OPC patients, including six XB type, three MS type and four IR type (Fig. 5a). Consistent with previous reports, the XB type showed high prevalence of *p53* mutations. The non-synonymous TMB was not different among subtypes. The non-synonymous TMB was not different among subtypes, and there was no correlation between TIDE score and TMB (Fig. 5b). One XB type tumour (YOPC31) had mutations in the DNA damage pathway (RAD50, RAD51, BLM and BRD7), with the highest mutational burden (6.24 mutations/Mb), but the TIDE score of this tumour indicated lower responsiveness to anti-PD-1/PD-L1 therapy. These findings are in line with a recent report of pan-cancer database analysis, showing a discrepancy between TMB and T cell-inflamed tumour signature.^23^ We also analysed the mutation signature of tumours defined by COSMIC database.^24^ The XB type showed enrichment of signature 1A (C > T, age related), 16 (T > C, aetiology unknown) and 18 (C > A), and MS type showed high proportion of signature 1A and 11 (C > T, Fig. 5c). Of note, the IR type tumours were particularly enriched for signature 2 (C > T, APOBEC related) and signature 7 (C > T, ultraviolet light related). Accordingly, we found upregulation of APOBEC family genes (*APOBEC3B*, *APOBEC3D* and *APOBECC3G*) in IR type tumours compared with that in other types (Fig. 5d). These results suggest that IR type tumours show a distinct genetic aetiology of APOBEC activation, in accordance with previous studies reporting correlation of HPV-positive tumours with APOBEC mutation signatures and high immunogenicity of tumours with APOBEC mutation signatures.^25^

### Immune microenvironment subtype predicts response to anti-PD-1/PD-L1 therapy

**Fig. 6:**
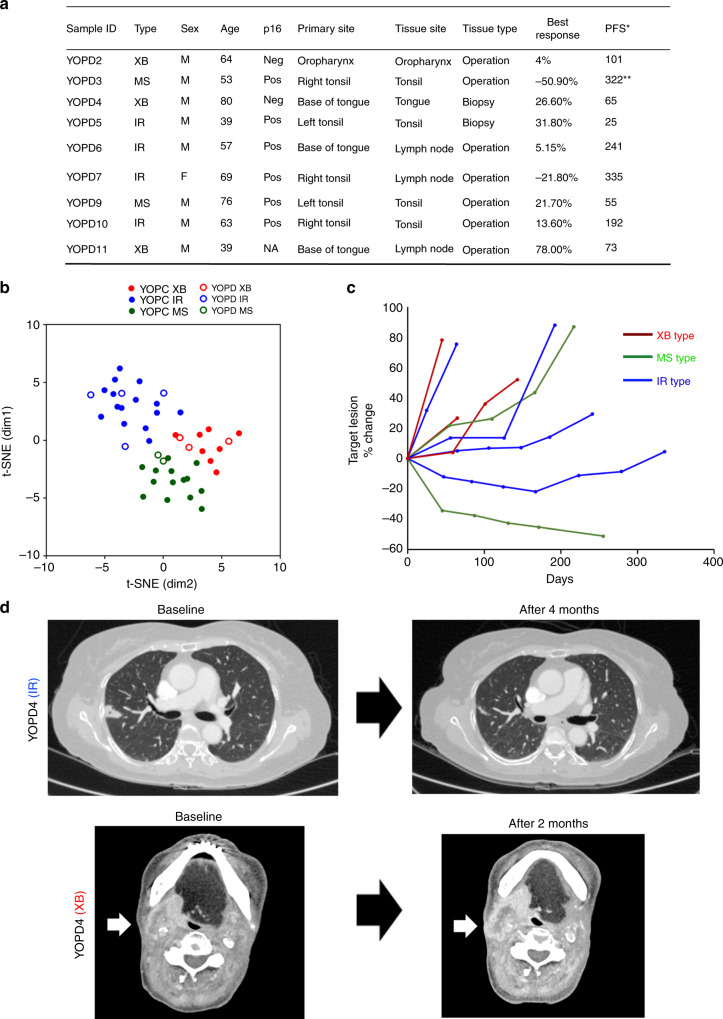
The molecular subtype predicts response to anti-PD-1/PD-L1 therapy in OPC patients. **a** The patient list and clinical characteristics of nine recurrent or metastatic OPC patients receiving anti-PD-1/PD-L1 therapy (YOPD). *The best response was determined by RECIST 1.1 criteria. **Patient still undergoing anti-PD-1/PD-L1 therapy. **b** The t-SNE plot for nine YOPD tumours combined with 37 YOPC tumours. The subtype of YOPD tumours was determined by distance from 37 YOPC tumours on the t-SNE plot. **c** Spider plot showing changes in target lesion diameters on anti-PD-1/PD-L1 therapy in nine OPC tumours according to subtype. **d** Representative CT scan images of IR type and XB type tumours on anti-PD-1/PD-L1 therapy.

We evaluated the predictive utility of the present OPC subtype classification in the response to anti-PD-1/PD-L1 therapy in OPC patients. We analysed pre-treatment tumour samples from nine patients with metastatic or recurrent OPC receiving anti-PD-1/PD-L1 therapy (YOPD, Fig. 6a). Employing t-SNE plotting combined with 37 surgically resected OPC tumours, we classified the nine YOPD tumours into four IR type, two MS type and three XB type tumours (Fig. 6b). Demonstrating the clinical significance of our subtyping, 75% of IR type tumours (3/4) showed tumour shrinkage (1/4) or tumour control (2/4) for more than 4 months upon anti-PD-1/PD-L1 therapy, whereas no XB type tumour showed clinical benefit from the anti-PD-1/PD-L1 therapy (Fig. 6c, d). Of the two MS type tumours, one showed a clinical benefit from anti-PD-1/PD-L1 therapy and the other did not. Although clinical significance of anecdotal findings stated above is limited by small sample size, these results suggest the potential poor response of XB type and favourable response of IR type OPCs to anti-PD-1/PD-L1 therapy.

## Discussion

The tumour microenvironment is based on competitive and cooperative interaction between tumour cells and tumour-infiltrating immune cells. Tumour cells evade immune responses by suppressing T lymphocyte functions and activating immune-suppressive cells. These immunologic dynamics influence the growth and metastasis of malignant tumours, as well as response to tumour immunotherapy. Here, we proposed molecular subtyping of OPCs to link the molecular aetiologies of tumours to their immune microenvironment properties. The three OPC subtypes, IR type, MS type and XB type, are related to adaptive immune response, MS response signatures and XB response signatures, respectively. Our analysis revealed distinct immunological profiles of each subtype that can be translated to diagnosis and treatment of OPC patients. We suggest that IR type tumours are strong responders to anti-PD-1/PD-L1 therapy with high immunogenicity, and the combination of anti-TGF-β therapy with immunotherapy would be useful for treating the MS type. The XB type exhibits potent innate resistance to anti-PD-1/PD-L1 therapy, and anti-CD73 treatment may provide therapeutic benefits in this subtype.

T cell-inflamed tumours show favourable survival outcomes in many cancer types,^26–28^ and a previous study showed a higher CD8^+^ T cell density in HPV-positive HNSCC tumours than in HPV-negative tumours.^12^ Moreover, evasion of the immune response against foreign HPV viral antigens in tumour cells is dependent on the PD-1/PD-L1 axis, suggesting that HPV-positive tumours are susceptible to the immune checkpoint pathway.^29^ Accordingly, the high T cell gene signature with positive HPV status of IR type tumours may explain the favourable prognosis and high responsiveness to anti-PD-1/PD-L1 therapy. However, it should also be noted that not all HPV-positive tumours were IR type, and a subset of HPV-positive tumours was classified as MS or XB type in our study. Previous studies indicated that HPV-positive HNSCC also involves downregulation of antigen-processing machineries,^30^ increased Treg infiltration^31^ and high IDO1 expression,^32^ suggesting that HPV-positive tumours are also dependent on various immune evasion mechanisms. Moreover, previous clinical trials demonstrated little difference in efficacy of anti-PD-1/PD-L1 therapy between HPV-positive and -negative HNSCCs.^7,8^ Our study implies that the immune response of OPC tumours is not determined solely by HPV status, as the immune microenvironment affects the anti-tumour immune response of OPC tumours.

In this study, a considerable proportion of tumours were classified as “MS” with high TGF-β activity. As demonstrated by a recent study, TGF-β signalling acts as the central node in the immune-exclusion phenotype, in which a high stromal component excludes T cells from tumour nests in bladder cancer models.^2^ We found a similar immune-exclusion mechanism in the MS type OPC tumours, as indicated by concomitant T cell exclusion and upregulation of f-TBRS. We expect that TGF-β signal inhibitors would be particularly beneficial in MS subtypes when combined with immune checkpoint blockade in clinical settings.

Results for targeted panel sequencing showed that the TMB was not significantly different among subtypes and that the TMB did not correlate with T cell infiltration signature. High TMB and T cell inflammation signature have been regarded as essential predictive biomarkers in immune checkpoint blockade therapy; however, a recent study revealed that these two markers have low correlation.^23^ Based on the present data, it is not clear whether high TMB XB type tumours are susceptible to anti-PD-1/PD-L1 therapy. However, our data suggest that immune-suppressive mechanisms, including the adenosine pathway with CD39/CD73 expression, may be active in XB type tumours. Our genomics analysis also revealed high APOBEC mutation signature in IR type tumours. A previous study pointed out that strong response to immunotherapy in non-small-cell lung cancer patients is related to high APOBEC signatures,^25^ and another reported high PD-1 expression in hypermutated bladder cancer tumours with APOBEC signatures.^33^ Therefore, the high immunogenicity of the IR type may be related to the high APOBEC activity in the tumour cells. Our results collectively suggest that the different immunologic properties of OPC subtypes originate from their different genetic aetiologies.

Although immune checkpoint blockades elicit long-term clinical benefit in responsive tumours, the proportion of responsive tumours is substantially limited in HNSCC. Appropriate patient selection is the key factor in tumour immunotherapy; however, previous studies have shown a discrepancy between immunotherapy response and PD-L1 IHC expression. We expect that the present subtype classification will be useful as a companion diagnostic tool for OPC patients in clinical settings. The development of convenient techniques, such as IHC or nanostring RNA detection, for determining OPC subtypes should facilitate effective immunotherapy in OPC patients. In the future, subtype-based combination therapy trials, such as combination of other immune checkpoint blockades (LAG3 or TIGIT with PD-1) in IR type, TGF-β inhibition in MS type and anti-CD73 therapy in XB type will likely broaden the scope of application of immunotherapy strategies in OPC cancer patients.

In this study, we thoroughly studied OPC tumours using a combination of RNA-seq, targeted sequencing and multiplex IHC analysis. However, our study has limitations because of its retrospective nature, and a limited sample size. The differential response of OPC subtypes to anti-PD-1/PD-L1 therapy should be interpreted with caution because only nine patients were analysed. Further studies are warranted to validate our findings. The proportion of tumour stage was also different among three subtypes (Supplementary Table S2), and the clinical implication of the difference needs to be investigated in another cohort.

In conclusion, our study firstly demonstrates distinct immune microenvironments in subtypes of OPC tumours and their clinical implications in patient prognosis and response to anti-PD-1/PD-L1 therapy. We suggest that the appropriate classification of tumour subtypes is an essential step towards effective immunotherapy in OPC patients. Our findings are expected to improve treatment outcomes by enabling reliable molecular diagnosis and combination immunotherapy.

## Supplementary information

supplementary methods and supplementary figure, tables

supplementary table 4

supplementary table 5

supplementary table 6

## Data Availability

The datasets used and/or analysed during the current study are available from the corresponding author on reasonable request.
