# Correction: Molecular subtypes of oropharyngeal cancer show distinct immune microenvironment related with immune checkpoint blockade response

**DOI:** 10.1038/s41416-020-0944-1

**Published:** 2020-06-22

**Authors:** Min Hwan Kim, Jae-Hwan Kim, Ji Min Lee, Jae Woo Choi, Dongmin Jung, Hojin Cho, Hyundeok Kang, Min Hee Hong, Su Jin Heo, Se Heon Kim, Eun Chang Choi, Da Hee Kim, Young Min Park, Sangwoo Kim, Sun Och Yoon, Yoon Woo Koh, Byoung Chul Cho, Hye Ryun Kim

**Affiliations:** 1 Department of Internal Medicine, Division of Medical Oncology, Yonsei Cancer Center, Yonsei University College of Medicine, Seoul, Republic of Korea; 2 Institute for Cancer Research, Yonsei Cancer Center, Yonsei University College of Medicine, Seoul, Republic of Korea; 3 Brain Korea 21 PLUS Project for Medical Science, Yonsei University College of Medicine, Seoul, Republic of Korea; 4 Department of Pharmacology, Yonsei University College of Medicine, Seoul, Republic of Korea; 5 Department of Nuclear Medicine, Yonsei University College of Medicine, Seoul, Republic of Korea; 6 Department of Biomedical Systems Informatics, Brain Korea 21 PLUS Project for Medical Science, Yonsei University College of Medicine, Seoul, Korea; 7 Department of Otorhinolaryngology, Yonsei University College of Medicine, Seoul, Republic of Korea; 8 Department of Pathology, Yonsei University College of Medicine, Seoul, Republic of Korea

**Keywords:** Tumour immunology, Cancer microenvironment, Head and neck cancer

Correction to: *British Journal of Cancer* (2020) **122**, 1649–1660; 10.1038/s41416-020-0796-8, published online 1 April 2020

Since the publication of this paper the authors noticed an error in the name of Sangwoo Kim, where Dr Kim’s name was incorrectly listed as Sang Woo Kim. This has been corrected above.

