# Supplementary material for: Molecular subtypes of oropharyngeal cancer show distinct immune microenvironment related with immune checkpoint blockade response
Source: Br J Cancer. 2020 Apr 1;122(11):1649–60. doi: 10.1038/s41416-020-0796-8 (PMC7251088; doi:10.1038/s41416-020-0796-8)
Supplement: Supplementary file 1 — supplementary methods and supplementary figure, tables [file 41416_2020_796_MOESM1_ESM.pdf]

Supplementary Information

Molecular subtypes of oropharyngeal cancer show distinct immune microenvironment related with immune checkpoint blockade response

Min Hwan Kim[1,†], Jae-Hwan Kim[2,†], Ji Min Lee[3], Jae Woo Choi[2,4], Dongmin Jung[2], Hojin Cho[5], Hyun Duk Kang[6], Min Hee Hong[1], Su Jin Heo[1], Se Heon Kim[7], Eun Chang Choi[7], Da Hee Kim[7], Young Min Park[7], Sang Woo Kim[6], Sun Och Yoon[8, ‡], Yoon Woo Koh[7, ‡], Byoung Chul Cho [1, ‡], Hye Ryun Kim [1, ‡, *]

-Contents-

1. Supplementary methods – page 2

2. Supplementary Figures – page 12

3. Supplementary Tables – page 16

1. Supplementary methods

Patients

This study analyzed two cohorts of patients who were diagnosed with squamous cell carcinoma of the oropharynx by pathologic confirmation at the Yonsei Cancer Center. The diagnosis of oropharyngeal (OPC) was performed by computed tomography or magnetic resonance imaging of the head and neck, and only tumors whose epicenter originated from the soft palate, the base of tongue, the tonsils, and the side and back wall of the throat were included in the study. The first cohort consisted of 37 patients with local or locally advanced OPC who received curative resection between January 2011 and December 2014. We collected the clinical and pathological data by electronic medical chart review, and survival outcome data were recorded. The 7th American Joint Committee on Cancer (AJCC) staging criteria were used for tumor staging. The second cohort included 9 recurrent or metastatic OPC patients who were administered anti-PD-1/PD-L1 blocking agents between December 2016 to October 2018. The changes in target lesions were measured according to Response evaluation criteria in solid tumors 1.1 (RECIST). This study was reviewed and approved by the Institutional Review Board of Severance Hospital.

Pathological review and HPV status

Formalin-fixed and paraffin-embedded (FFPE) tumor specimens were obtained from all patients in the two patient cohorts. Tumor specimens obtained by surgery of primary tumors were collected from 37 surgically resected OPC patients. In addition, pre-treatment archival FFPE specimens, which were either biopsy or surgery specimens, were collected from 9 anti-PD-1/PD-L1-treated patients. The diagnosis of squamous cell carcinoma of the oropharynx was confirmed by examining hematoxylin and eosin stained slides. The HPV status of the OPC

tumors was determined by IHC staining of p16 protein in tumor cells. The HPV status of 37 surgically resected tumors was also evaluated by Anyplex™ II HPV28 Detection Kit (Seegene) according to the manufacturer's instructions using tumor DNA extracted from FFPE tumor specimens.

RNA-seq experiment

The RNA-seq was performed using AmpliSeq panel and Ion Torrent platform for 37 surgically resected OPC tumors (YOPC) as described previously,[1] and TruSeq RNA Access library kit and HiSeq 2500 (Illumina, San Diego, USA by the Macrogen Incorporated) for 10 OPC tumors treated with anti-PD-1/PD-L1 therapy. For 37 surgically resected OPC tumor samples, 10 ng of total RNA was reverse transcribed to cDNA using the Ion AmpliSeq Transcriptome Human Gene Expression Kit (Life Technologies, Carlsbad, CA, USA). Analysis of the Ion Proton reads was performed using the AmpliSeqRNA analysis plugin, v4.2.1, in Torrent Suite Software (Life Technologies, Carlsbad, CA, USA), which is designed to count the number of sequences obtained from cDNA amplicons. From these resulting counts, the AmpliSeq Human Gene Expression panel was used to measure expression levels of over 20,800 different genes.[2]

For 9 OPC tumors treated with anti-PD-1/PD-L1 therapy (YOPD), RNA sequencing libraries were prepared using TruSeq RNA Access library kit (Illumina, Inc., San Diego, CA, USA). cDNA syntheses were performed using SuperScript II Reverse Transcriptase (Thermo Fisher Scientific Inc., Waltham, MA, USA) and PCR amplification after performing adaptor ligation. After validation of the libraries with Agilent DNA screentape D1000 kit on a TapeStation (Agilent Technologies, Santa Clara, CA, USA), the first hybridization step was performed using exome capture probes. Then, streptavidin-coated magnetic beads were used to capture probes hybridized to the target regions. The enriched libraries were amplified by second PCR

and the final libraries were quantitated by qPCR using KAPA Library Quantification Kit—Illumina/ABI Prism® (Kapa Biosystems, Inc., Wilmington, MA, USA) and validated by Agilent DNA screentape D1000 kit on a TapeStation (Agilent Technologies, Santa Clara, CA, USA). The libraries were then sequenced using HiSeq 2500 (Illumina, San Diego, USA by the Macrogen Incorporated) platform.

RNA-seq data analysis

Trimming was performed to remove adapters from the RNA sequence reads using CutAdapt. [3] Alignment and quantification of trimmed reads were performed using RSEM. [4] For different platforms, batch effects were corrected by the ComBat algorithm from surrogate variable analysis (SVA) package. DESeq2 package was used for differentially expressed gene (DEG) analysis. The RNA-seq data from OPC tumors were subjected to two-dimensional t-Distributed Stochastic Neighbor Embedding (t-SNE) plotting using Rtsne package, after filtering out genes with low expression (sum of normalized count across samples <10 or expressed in less than 5 samples). Unsupervised clustering of the tSNE result was performed using k-means clustering. The clustering yielded three molecular subtypes of OPC tumors. The subtype-specific gene signatures were established by DEG analysis upon comparing each subtype to other two subtypes (log fold change ≥ 2 and *P*-value <0.05). The 53 genes for CL type, 397 genes for MS type, and 144 genes for IR type were defined as subtype-specific gene signatures.

Molecular subtyping in TCGA cohort

RNA-seq data for a previous TCGA study on HNSCC was downloaded, and the OPC patients (n=33) with tonsil, base of tongue, and oropharynx tumor origins were selected. The molecular subtypes of TCGA OPC were determined by tSNE plotting and k-means clustering in the

combined cohort of YCC OPC and TCGA OPC patients (n = 70). RNA-seq data for tumors from 518 HNSCC patients in the TCGA database were also downloaded using Firebrowse (Broad Institute) in the form of RSEM normalized count. The enrichment score for each subtype-specific gene signature (CL, MS, and IR type gene signature) was also calculated using gene set variation analysis (GSVA) package in 518 HNSCC tumors. The tumors were classified into CL, MS, and IR types based on k-means clustering of enrichment score of each subtype, and their overall survival was compared by obtaining Kaplan-Meier curves using the log-rank test.

Molecular characterization of subtypes

The DEGs among three the subtypes with cutoff of log fold change ≥ 1 were used for molecular characterization of each subtype. The six gene clusters were identified by hierarchical clustering of DEGs in GENE-E (Broad institute) according to their expression in each subtype. The gene ontologies of the six gene clusters were analyzed using DAVID software, and top-enriched ontology terms were used to determine the characteristics of the six clusters. The gene expression level on z-score transformation in each subtype was compared (Figure 2B). GSVA analysis of RNA-seq data using HALLMARK signatures (H) in MSigDB (Broad Institute) was also performed to identify enriched gene signatures in each subtype. Network analysis was performed on pair-wise compared DEGs of each subtype using Enrichment map software [5].

Analysis of immunologic properties of molecular subtypes using RNA-seq data

To examine the gene expression of immune-related genes, the immune response-related genes (from GO term "immune response", GO:0006955) were selected from the DEGs among the three subtypes. (595 genes, log2 Fold change ≥ 1 and P-value <0.05). The expression of genes

related to T cell exhaustion and macrophage/granulocyte infiltration and activation was compared. The gene set enrichment analysis (GSEA, Broad Institute) was also performed to analyze enrichment of T cell exhaustion signatures (LAYN16 and Mel7517) and myeloid cell- or macrophage-related signatures (GO term "Myeloid leukocyte mediated immunity"/GO:0002444, GO term "Regulation of macrophage activation"/GO:0043030).

The CIBERSORT algorithm was used for deconvolution of RNA-seq data to estimate the composition of tumor-infiltrating immune cells.[6] The proportion of immune cells in each tumor was compared according to subtypes. The Tumor Immune Dysfunction and Exclusion (TIDE) score[7] was calculated for each tumor to predict favorable response to anti-PD-1/PD-L1 therapy. The GSVA enrichment score for the previously reported 19 signatures related to innate anti-PD-1 resistance (IPRES) was also calculated.[8]

Targeted panel sequencing

Genomic DNA was purified from FFPE tissues and peripheral blood mononuclear cells from patients' blood using the QIAamp DNA FFPE Tissue Kit (Qiagen, Hilden, Germany) for targeted sequencing. As previously described,[9] the 244 head and neck cancer-related genes were incorporated into our targeted sequencing panel. To capture the genomic regions of these genes, the customized SureSelectXT Target Enrichment library generation kit (Agilent, Santa Clara, CA, USA) was used. These regions were sequenced using the Illumina HiSeq 2500 platform with a depth of coverage > 1000×. Next, quality-based trimming with Sickle [10] was performed for short reads from the targeted sequencing. Using Burrows-Wheeler Aligner,[11] filtered reads were mapped to the human reference genome (GRCh37/hg19) and those with <20 mapping quality were discarded. The aligned reads (BAM file) were further processed using the Genome Analysis ToolKit v3.5 with MarkDuplicate, LocalRealignment, and

BaseQualityScoreRecalibration.[12] A set of somatic mutations was called by MuTect ver. 1.17 with default parameters.[13] Somatic insertions/deletions were detected by Scalpel with default parameters.[14] Each variant was annotated by ANNOVAR in terms of mutation consequences, predicted impacts, and reported allele frequencies in normal population.[15] Oncoprint was drawn from somatic variants using the R package 'ComplexHeatmap'[16] for visualization of the overall landscape of variants. Mutational signatures of head and neck cancer were analyzed using the R package 'deconstructSig'.[17] The tumor mutational burden (TMB), defined as the number of missense mutations per Mb from the targeted sequencing data, which has 1,122,264 base pair of target region. The TMB was compared among subtypes of OPC tumors.

Multiplex immunohistochemistry (IHC) image analysis

For multiplex IHC analysis, 4-μm-thick paraffin tissue sections were generated from FFPE tumor blocks. Tissue sections mounted on slides were stored in the dark at room temperature, which were stained within 4 months of sectioning to preserve antigenicity. For multiplex immunofluorescence stain of the sections, the automated staining system (BOND Rx, Leica Biosystems) was used with the Opal 7-color automation IHC kit (PerkinElmer). All procedure was followed by manufacturer's instructions. In summary, after deparaffinized sections were incubated with citrate- or Tris-based antigen unmasking solutions at 100ºC for 20 min, the primary antibody, HRP-conjugated antibody and fluorophore were consequently applied to the sections. The primary antibodies were CD8 [SP16], CD68 [D4B9C], CD 73 [D7F9A], PD-1 [EPR4877(2)], PD-L1 [22C3], pan-cytokeratin (CK) [AE1/AE3] and p63 [A4A], and 4′,6-diamidino-2-phenylindole (Spectral DAPI, PerkinElmer) was used for counter stain. The multi-spectral images (200 x magnification) for analyses of tissue contents were generated from whole-slide scan images by Vectra polaris (PerkinElmer) and Phenochart software

(PerkinElmer). To determine the tumor nest and stromal region, the sites positive or negative for the pan-CK/p63 stain were categorized by the integral algorithm in InFrom 2.0 software (PerkinElmer), which were confirmed with H&E-stained slides. Immune cells were quantified by cell counts per mm2 in both the tumor nest and stroma. Membranous PD-L1 or CD73 expression on tumor cells was determined by Tumor Proportion Score (TPS) in InForm software.

PET-CT imaging and analysis

All patients underwent F-18 FDG PET/CT on either a Biograph Truepoint 40 PET/CT scanner (Siemens Healthcare, Erlangen, Germany) or a Discovery STe PET/CT scanner (GE Healthcare, Milwaukee, WI). Patients fasted for at least 6 h before scans, and peripheral blood glucose levels were not higher than 140 mg/dL before F-18 FDG injection. Approximately 5.5 MBq of F-18 FDG per kilogram of body weight was administered intravenously 1 h before the start of imaging. After the initial low-dose CT (Biograph TruePoint 40: 36 mA, 120 kVp; Discovery STe: 30 mA, 140 kVp), standard PET imaging was conducted from the cerebellum to the mid-thigh, with acquisition times of 2.5 min/bed position for the Biograph TruePoint 40 scanner and 3 min/bed position for the Discovery STe scanner in 3-dimensional mode. PET images were reconstructed iteratively with CT-based attenuation correction.

All F-18 FDG PET/CT images were reviewed by a nuclear medicine physician using MIM imaging software (MIM 6.5; MIM Software Inc, Cleveland, OH). The SUV_{max} values were measured in a volume of interest (VOI) drawn on PET images. In each patient, the SUV_{max} of the primary tumor was measured. Normal background liver SUV_{mean} values of the SUV were measured by drawing three 1-cm-sized VOIs in the liver: two in the right lobe and one in the

left lobe. The SUV of the VOI was calculated as follows: decay-corrected activity (kilobecquerel) per milliliter of tissue volume/injected F-18 FDG activity (kilobecquerel)/body weight (gram). The SUV_{max} of the primary tumor was divided by the SUV_{mean} of the background liver to yield TLR. We normalized the SUV_{max} by the SUV_{mean} of the background liver to compensate for the use of different scanner types.

Statistical analysis

The clinical and pathologic characteristics among three molecular subtypes were compared using Fisher's exact test for categorical variables and Kruskall-Wallis test for continuous variables. The survival difference among three subtypes was compared by Kaplan-Meier plots. The other experimental results were compared using unpaired Student's t-test. Data analysis was performed using GraphPad Prism (GraphPad Software), and statistical significance was considered when p-value was <0.05 in an unpaired Student's t-test (* p<0.05; ** p<0.01).

References

1. Kim HR, Ha SJ, Hong MH, Heo SJ, Koh YW, Choi EC, Kim EK, Pyo KH, Jung I, Seo D, Choi J, Cho BC, Yoon SO (2016) PD-L1 expression on immune cells, but not on tumor cells, is a favorable prognostic factor for head and neck cancer patients. *Sci Rep* **6**: 36956.
2. Osman A, Hitzler WE, Ameur A, Provost P (2015) Differential Expression Analysis by RNA-Seq Reveals Perturbations in the Platelet mRNA Transcriptome Triggered by Pathogen Reduction Systems. *PLoS One* **10**(7): e0133070.
3. Martin M (2011) Cutadapt removes adapter sequences from high-throughput sequencing reads. *2011* **17**(1): 3.
4. Li B, Dewey CN (2011) RSEM: accurate transcript quantification from RNA-Seq data with or without a reference genome. *BMC Bioinformatics* **12**: 323.
5. Merico D, Isserlin R, Stueker O, Emili A, Bader GD (2010) Enrichment map: a network-based method for gene-set enrichment visualization and interpretation. *PLoS One* **5**(11): e13984.
6. Newman AM, Liu CL, Green MR, Gentles AJ, Feng W, Xu Y, Hoang CD, Diehn M, Alizadeh

AA (2015) Robust enumeration of cell subsets from tissue expression profiles. *Nat Methods* **12**(5): 453-457.

7. Jiang P, Gu S, Pan D, Fu J, Sahu A, Hu X, Li Z, Traugh N, Bu X, Li B, Liu J, Freeman GJ, Brown MA, Wucherpfennig KW, Liu XS (2018) Signatures of T cell dysfunction and exclusion predict cancer immunotherapy response. *Nat Med* **24**(10): 1550-1558.
8. Hugo W, Zaretsky JM, Sun L, Song C, Moreno BH, Hu-Lieskovan S, Berent-Maoz B, Pang J, Chmielowski B, Cherry G, Seja E, Lomeli S, Kong X, Kelley MC, Sosman JA, Johnson DB, Ribas A, Lo RS (2016) Genomic and Transcriptomic Features of Response to Anti-PD-1 Therapy in Metastatic Melanoma. *Cell* **165**(1): 35-44.
9. Lim SM, Cho SH, Hwang IG, Choi JW, Chang H, Ahn MJ, Park KU, Kim JW, Ko YH, Ahn HK, Cho BC, Nam BH, Chun SH, Hong JH, Kwon JH, Choi JG, Kang EJ, Yun T, Lee KW, Kim JH, Kim JS, Lee HW, Kim MK, Jung D, Kim JE, Keam B, Yun HJ, Kim S, Kim HR (2018) Investigating the Feasibility of Targeted Next-Generation Sequencing to Guide the Treatment of Head and Neck Squamous Cell Carcinoma. *Cancer Res Treat* doi:10.4143/crt.2018.012.
10. Joshi NA, Fass JN. (2011). Sickle: A sliding-window, adaptive, quality-based trimming tool for FastQ files

(Version 1.33) [Software]. Available at https://github.com/najoshi/sickle. In.

11. Li H, Durbin R (2009) Fast and accurate short read alignment with Burrows-Wheeler transform. *Bioinformatics* **25**(14): 1754-1760.
12. McKenna A, Hanna M, Banks E, Sivachenko A, Cibulskis K, Kernytsky A, Garimella K, Altshuler D, Gabriel S, Daly M, DePristo MA (2010) The Genome Analysis Toolkit: a MapReduce framework for analyzing next-generation DNA sequencing data. *Genome Res* **20**(9): 1297-1303.
13. Cibulskis K, Lawrence MS, Carter SL, Sivachenko A, Jaffe D, Sougnez C, Gabriel S, Meyerson M, Lander ES, Getz G (2013) Sensitive detection of somatic point mutations in impure and heterogeneous cancer samples. *Nat Biotechnol* **31**(3): 213-219.
14. Fang H, Bergmann EA, Arora K, Vacic V, Zody MC, Iossifov I, O'Rawe JA, Wu Y, Jimenez Barron LT, Rosenbaum J, Ronemus M, Lee YH, Wang Z, Dikoglu E, Jobanputra V, Lyon GJ, Wigler M, Schatz MC, Narzisi G (2016) Indel variant analysis of short-read sequencing data with Scalpel. *Nat Protoc* **11**(12): 2529-2548.
15. Wang K, Li M, Hakonarson H (2010) ANNOVAR: functional annotation of genetic variants from high-throughput sequencing data. *Nucleic Acids Res* **38**(16): e164.
16. Gu Z, Eils R, Schlesner M (2016) Complex heatmaps reveal patterns and correlations in multidimensional genomic data. *Bioinformatics* **32**(18): 2847-2849.
17. Rosenthal R, McGranahan N, Herrero J, Taylor BS, Swanton C (2016) DeconstructSigs: delineating mutational processes in single tumors distinguishes DNA repair deficiencies and patterns of carcinoma evolution. *Genome Biol* **17**: 31.
18. Walter V, Yin X, Wilkerson MD, Cabanski CR, Zhao N, Du Y, Ang MK, Hayward MC, Salazar

AH, Hoadley KA, Fritchie K, Sailey CJ, Weissler MC, Shockley WW, Zanation AM, Hackman T, Thorne LB, Funkhouser WD, Muldrew KL, Olshan AF, Randell SH, Wright FA, Shores CG, Hayes DN (2013) Molecular subtypes in head and neck cancer exhibit distinct patterns of chromosomal gain and loss of canonical cancer genes. *PLoS One* **8**(2): e56823.

2. Supplementary Figures

Supplementary Figure S1. Comparison of our subtype classification with TCGA subtypes.

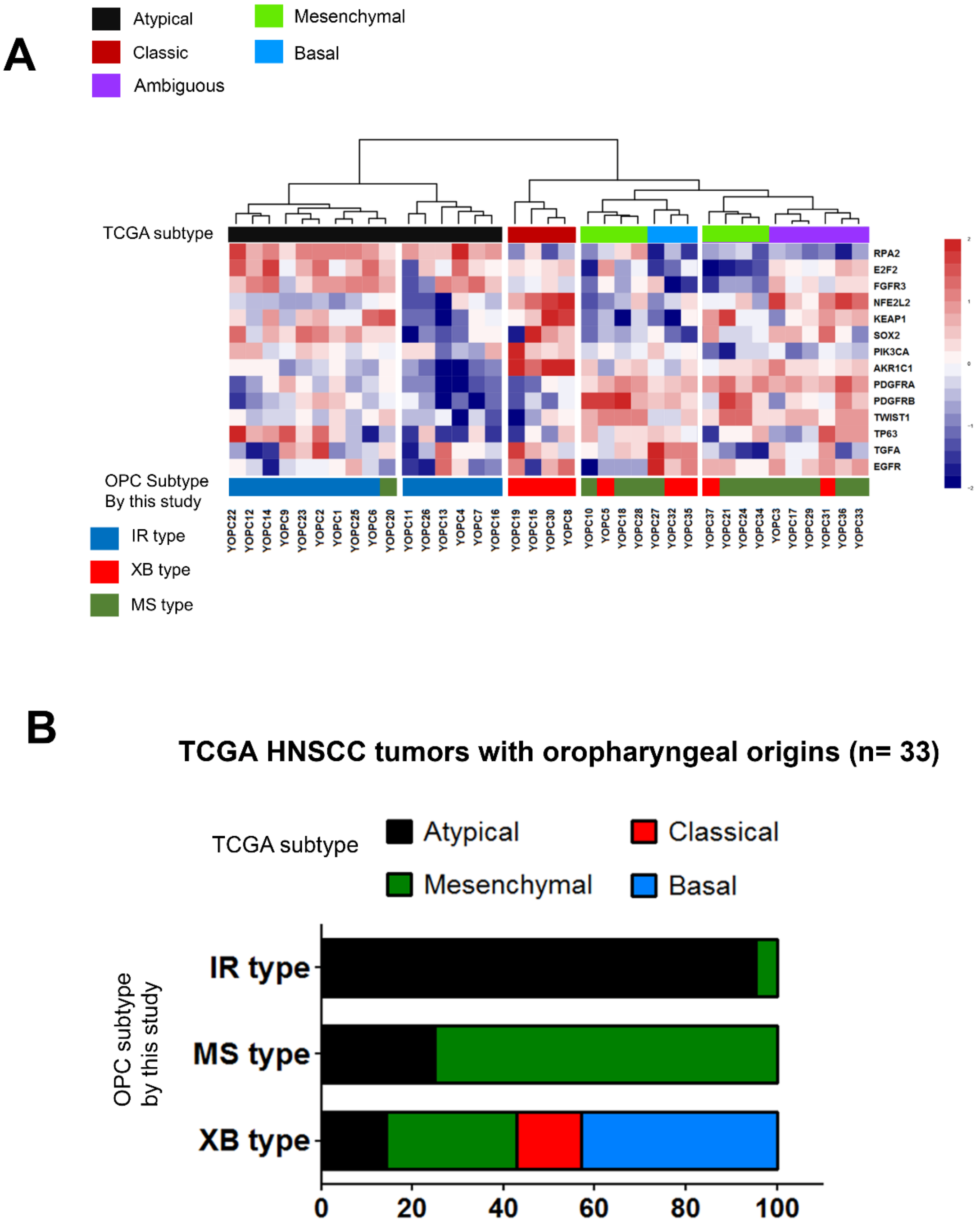

(A) Unsupervised hierarchical clustering analysis of our 37 surgically resected OPC samples (YOPC) using 14 representative genes for subtyping used in TCGA analysis (Walter et al., 2013[18]). The clusters were classified as TCGA subtypes (upper) based on gene expressions. Our tSNE clustering results were described below (lower)

(B) Comparison of TCGA subtypes and our tSNE subtypes in 33 TCGA HNSCC tumors with oropharyngeal origins.

Supplementary Figure S2. Comparison between GSVA subtype and tSNE subtypes.

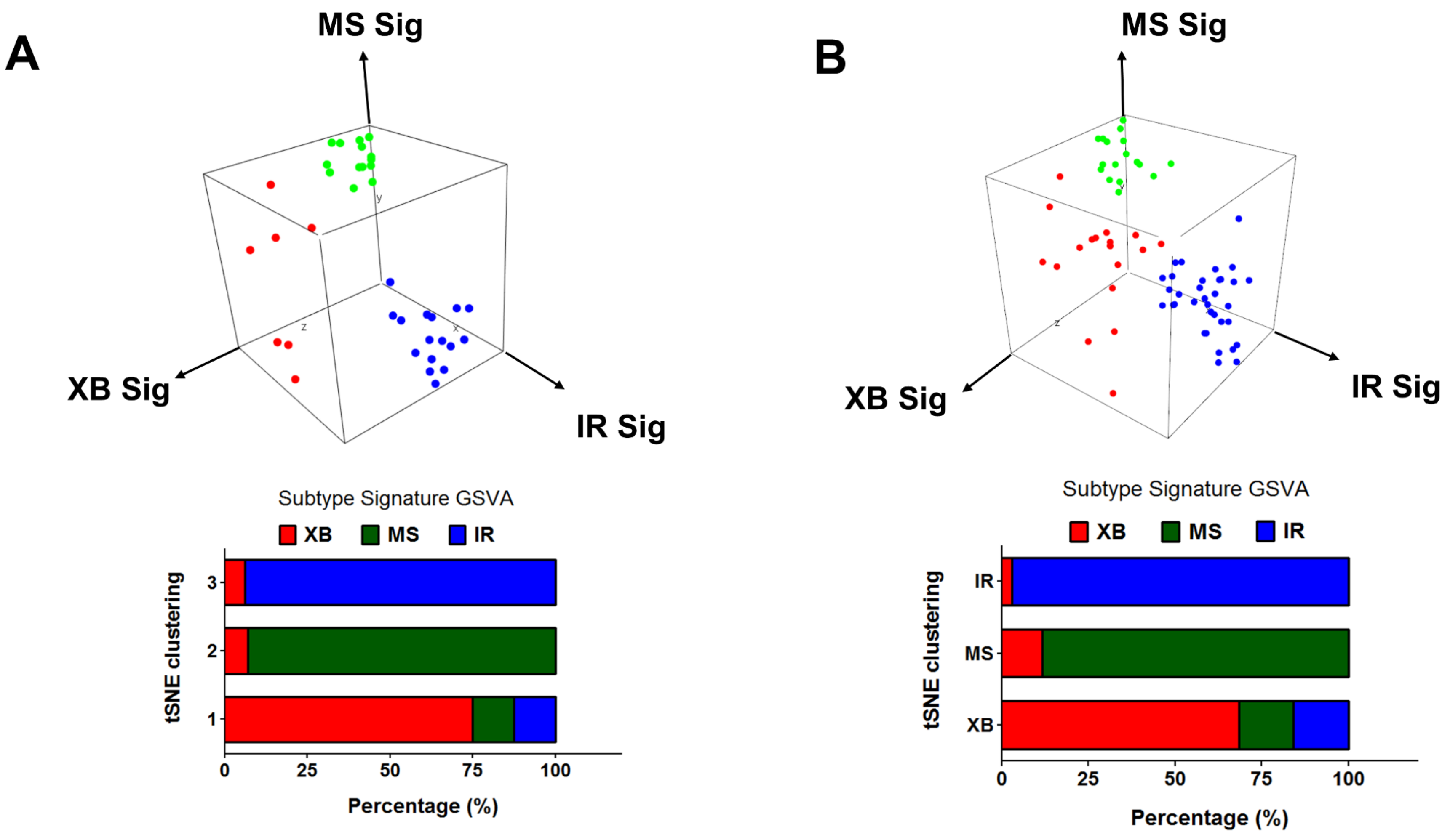

The three-dimensional plotting and k-means clustering analysis using GSVA score of each subtype signature derived from Figure 1E (upper panel) and comparison between GSVA clustering classification and tSNE plotting classification described in Figure 1A (lower panel)

(A) The comparison in 37 surgically resected OPC samples (YOPC)

(B) The comparison in combined cohort tumors (37 YOPC + 33 TCGA OPC tumors)

.

3. Supplementary Tables

Supplementary Table S1. Comparison of HPV PCR and p16 IHC in of 37 surgically resected OPC tumors (YOPC)

Tumor number	cluster_HPV	HPV PCR result	p16 IHC
YOPC01	IR	HPV DNA+	Positive
YOPC02	IR	HPV DNA+	Positive
YOPC04	IR	HPV DNA+	Positive
YOPC06	IR	HPV DNA+	Positive
YOPC07	IR	HPV DNA+	Positive
YOPC09	IR	HPV DNA+	Positive
YOPC11	IR	HPV DNA+	Positive
YOPC12	IR	HPV DNA+	Positive
YOPC13	IR	HPV DNA+	Positive
YOPC14	IR	HPV DNA+	Positive
YOPC16	IR	HPV DNA+	Positive
YOPC22	IR	HPV DNA+	Positive
YOPC23	IR	HPV DNA+	Positive
YOPC25	IR	HPV DNA+	Positive
YOPC26	IR	HPV DNA+	Positive
YOPC03	MS	Negative	Negative
YOPC10	MS	HPV DNA+	Positive
YOPC17	MS	Negative	Positive
YOPC18	MS	HPV DNA+	Positive
YOPC20	MS	HPV DNA+	Positive
YOPC21	MS	HPV DNA+	Positive
YOPC24	MS	HPV DNA+	Negative
YOPC27	MS	Negative	Negative
YOPC28	MS	Negative	Negative
YOPC29	MS	Negative	Negative
YOPC33	MS	Negative	Negative
YOPC34	MS	Negative	Negative
YOPC36	MS	Negative	Negative
YOPC37	MS	HPV DNA+	Negative
YOPC05	XB	HPV DNA+	Positive
YOPC08	XB	Negative	Negative
YOPC15	XB	Negative	Negative
YOPC19	XB	Negative	Negative
YOPC30	XB	Negative	Negative
YOPC31	XB	Negative	Negative
YOPC32	XB	Negative	Negative
YOPC35	XB	Negative	Negative

Supplementary Table S2. Baseline characteristics of OPC patients according to subtype

		IR type (n=15)	MS type (n=14)	XB type (n=8)	P-value*
**Age**	**Median (IQR)**	58 (48~64)	61.5 (56~71)	67 (55.5~74)	0.292
**Sex**	**Female**	4 (26.7%)	3 (21.4%)	0 (0%)	0.373
	**Male**	11 (73.3%)	11 (78.6%)	8 (100%)	
**Smoking (PYRS)**	**Median (IQR)**	10 (0~23)	32 (0~40)	37 (29~48)	**0.028**
**Primary tumor sites**	**BOT**	1 (6.7%)	4 (28.6%)	1 (12.5%)	0.088
	**Oropharynx**	1 (6.7%)	1 (7.1%)	0 (0%)	
	**Soft palate**	0 (0%)	1 (7.1%)	0 (0%)	
	**Tonsil**	13 (86.7%)	8 (57.1%)	5 (62.5%)	
	**Uvula**	0 (0%)	0 (0%)	2 (25.0%)	
**Alcohol**	**Yes**	8 (53.3%)	7 (50%)	5 (62.5%)	0.915
**Stage**	**I**	0 (0%)	6 (42.9%)	1 (12.5%)	**0.023**
	**II**	2 (13.3%)	0 (0%)	1 (12.5%)	
	**III**	1 (6.7%)	2 (14.3%)	0 (0%)	
	**IVA**	12 (80%)	6 (42.9%)	6 (75%)	
**PNI**	**Yes**	1 (6.7%)	1 (7.1%)	2 (25%)	0.415
**LVI**	**Yes**	5 (33.3%)	2 (14.3%)	3 (37.5%)	0.444
**Resection margin**	**Positive**	3 (20%)	2 (14.3%)	3 (37.5%)	0.516
**Differentiation**	**WD**	0 (0%)	3 (21.4%)	0 (0%)	0.179
	**MD**	7 (46.7%)	7 (50%)	6 (75%)	
	**PD**	8 (53.3%)	4 (28.6%)	2 (25%)	
**Recurrence**	**Yes**	1 (6.7%)	1 (7.1%)	4 (50%)	**0.016**

*p-values were calculated using Fisher's exact test for categorical variables, and Mann-Whitney U test for continuous variables

Abbreviations: IR, immune-rich; MS, mesenchymal; XB, xenobiotic; IQR, interquartile range; PYRS, pack-years; BOT, base of tongue; PNI, perineural invasion; LVI, lymphovascular invasion; WD, well-differentiated; MD, moderately-differentiated; PD, poorly-differentiate

Supplementary Table S3. Baseline characteristics of OPC patients according to subtype stratified by HPV status

		All patients			IR type	MS type		XB type	
		HPV neg (n=16)	HPV pos (n=21)	P-value	HPV pos (n=15)	HPV neg (n=9)	HPV pos (n=5)	HPV neg (n=7)	HPV pos (n=1)
Age		63.5 (57.5~73)	58 (48~63)	0.083	58 (48~64)	61 (58~72)	62 (56~63)	70 (57~77)	45 (45~45)
Sex	F	2 (12.5%)	5 (23.8%)	0.674	4 (26.7%)	2 (22.2%)	1 (20%)	0 (0%)	0 (0%)
	M	14 (87.5%)	16 (76.2%)		11 (73.3%)	7 (77.8%)	4 (80%)	7 (100%)	1 (100%)
Smoking (PYRS)		34 (22~50)	20 (0~36)	0.056	10 (0~23)	24 (0~50)	40 (30~40)	40 (33~55)	22 (22~22)
Alcohol	0	8 (50%)	9 (42.9%)	0.746	7 (46.7%)	6 (66.7%)	1 (20%)	2 (28.6%)	1 (100%)
	1	8 (50%)	12 (57.1%)		8 (53.3%)	3 (33.3%)	4 (80%)	5 (71.4%)	0 (0%)
Stage	I	7 (43.8%)	0 (0%)	0.001	0 (0%)	6 (66.7%)	0 (0%)	1 (14.3%)	0 (0%)
	II	1 (6.3%)	2 (9.5%)		2 (13.3%)	0 (0%)	0 (0%)	1 (14.3%)	0 (0%)
	III	0 (0%)	3 (14.3%)		1 (6.7%)	0 (0%)	2 (40%)	0 (0%)	0 (0%)
	IVA	8 (50%)	16 (76.2%)		12 (80%)	3 (33.3%)	3 (60%)	5 (71.4%)	1 (100%)
PNI	0	14 (87.5%)	19 (90.5%)	1.000	14 (93.3%)	9 (100%)	4 (80%)	5 (71.4%)	1 (100%)
	1	2 (12.5%)	2 (9.5%)		1 (6.7%)	0 (0%)	1 (20%)	2 (28.6%)	0 (0%)
LVI	0	14 (87.5%)	13 (61.9%)	0.137	10 (66.7%)	9 (100%)	3 (60%)	5 (71.4%)	0 (0%)
	1	2 (12.5%)	8 (38.1%)		5 (33.3%)	0 (0%)	2 (40%)	2 (28.6%)	1 (100%)
Resection margin	0	13 (81.3%)	16 (76.2%)	1.000	12 (80%)	8 (88.9%)	4 (80%)	5 (71.4%)	0 (0%)
	1	3 (18.8%)	5 (23.8%)		3 (20%)	1 (11.1%)	1 (20%)	2 (28.6%)	1 (100%)
Differentiation	WD	2 (12.5%)	1 (4.8%)	0.607	0 (0%)	2 (22.2%)	1 (20%)	0 (0%)	0 (0%)
	MD	9 (56.3%)	11 (52.4%)		7 (46.7%)	4 (44.4%)	3 (60%)	5 (71.4%)	1 (100%)
	PD	5 (31.3%)	9 (42.9%)		8 (53.3%)	3 (33.3%)	1 (20%)	2 (28.6%)	0 (0%)
Recurrence	0	14 (87.5%)	20 (95.2%)	0.568	15 (100%)	8 (88.9%)	5 (100%)	6 (85.7%)	0 (0%)
	1	2 (12.5%)	1 (4.8%)		0 (0%)	1 (11.1%)	0 (0%)	1 (14.3%)	1 (100%)

*p-values were calculated using Fisher's exact test for categorical variables, and Mann-Whitney U test for continuous variables. The p-value testing HPV positive versus HPV negative was not calculated due to small sample size

Abbreviations: IR, immune-rich; MS, mesenchymal; XB, xenobiotic; IQR, interquartile range; PYRS, pack-years; PNI, perineural invasion; LVI, lymphovascular invasion; WD, well-differentiated; MD, moderately-differentiated; PD, poorly-differentiate

Supplementary Table S4. Subtype-specific gene signatures by analyzing the differentially expressed genes (DEGs) of each subtype

Separate file

Supplementary Table S5. The significantly altered genes among subtypes classified into cluster 1 to 6 by hierarchical clustering
Separate file

Supplementary Table S6. Significantly altered immune response-related among the three subtypes
Separate file
